# Analysis of Rainfall Infiltration Stability of High Liquid Limit Red Clay Roadbed in Africa

**DOI:** 10.1155/2022/8222075

**Published:** 2022-01-21

**Authors:** Xiao Song, Hong Li

**Affiliations:** School of Transportation and Logistics Engineering, Wuhan University of Technology, Wuhan 430063, China

## Abstract

The annual rainfall in tropical rain forests in Africa is concentrated, and the abundant rainfall can easily lead to roadbed landslides. Therefore, it is necessary to analyze the impact of rainfall on the stability of roadbeds. This paper first uses the pore fluid permeability/stress coupling analysis step provided by ABAQUS to calculate the impact of rainfall infiltration on the overall stability of the roadbed slope and then discusses the rainfall infiltration on the slope seepage field, stress field, and displacement combined with the strength reduction method and the influence of field and safety factors. In the end, it is concluded that the 72-hour rainfall with an intensity of 50 mm/d will reduce the safety factor of the roadbed by 4.9% compared with before the rainfall. At the same time, it will increase the internal pore water pressure of the roadbed, reduce the suction of the matrix, and reduce the effective stress, which is caused by various factors. The overall stability of the roadbed is reduced.

## 1. Introduction

The Yadu expressway project is the most convenient fast track connecting Cameroon's political capital Yaoundé and the economic capital Douala, and it is also an important part of the cargo transshipment channel for inland countries such as Central Africa. Most of the project is high liquid limit red clay. According to the test data of Zhu et al. [1], the liquid limit of red clay is 60%, the plasticity index is 29, the particle diameter is less than 0.075 mm, and the content of fine particles is greater than 75%, which is classified as MH high liquid limit red clay. It is characterized by high natural moisture content and poor water stability. At the same time, the terrain of the project area fluctuates greatly, which belongs to tropical rain forest climate and rich rainfall. Rainfall concentration is a common natural phenomenon and one of the main factors causing slope instability, which poses a great hidden danger to the safety of people's lives and property. In the general subgrade design, we must pay special attention to the impact of rainfall on subgrade stability. In the meanwhile, the effect of the landslides which affect the safety and use efficiency of highway subgrade should be avoided. Therefore, it is of great importance to study the influence of rainfall infiltration on the internal seepage field and stress field of the red clay roadbed slope.

Fredlund and Anderson [2] applied the Galerkin finite element simulation method to slope seepage analysis under heavy rain conditions and found that negative pore water pressure can effectively increase the shear strength of soil. After using numerical simulation to study typical unsaturated slopes in Hong Kong, Wu et al. [3] obtained the factors that have a significant impact on slope stability, including the permeability coefficient, rainfall duration, and rainfall intensity. Yao et al. [4] found that there is a strong correlation between the slope stability and soil permeability coefficient under rainfall conditions.

When the rain intensity is less than the soil permeability coefficient, rainwater will continue to infiltrate, which will cause a continuous decrease in the shear strength of the roadbed; when the rain is stronger than the soil permeability coefficient, the change in rain intensity will have little effect on it. Therefore, this paper takes the high liquid limit red clay roadbed along the project as the research object and also combines with the laboratory test data to conduct a fluid-solid coupling analysis of rainfall infiltration in an attempt to find the safety factor, stress field, and seepage of the roadbed under different rainfall conditions. The related laws of field and displacement field provide references for slope prevention and mitigation.

## 2. ABAQUS Calculation Theory and Method

ABAQUS is a finite element simulation software with convenient operation, simple interactive interface, and excellent processing of nonlinear problems. The software specifically provides an analysis step for dealing with fluid-solid coupling problems so as to solve the seepage problem of saturated or unsaturated soil. ABAQUS can provide users with a general analysis step, which can perform linear or nonlinear analysis. The soil consolidation analysis, which is the soil analysis step, is just suitable for the seepage analysis in this paper. Through the analysis steps provided by the software, the process of rainfall infiltration can be well simulated. In the postprocessing module, the software can directly see the change process of slope safety factor, stress field, seepage field, and displacement field through various forms of cloud images, which is convenient to draw regular conclusions.

### 2.1. Saturated-Unsaturated Soil Seepage Control Equation

Darcy's law in classical saturated soil mechanics is based on the assumption of laminar flow in sand. It is used to describe the law of the functional relationship between seepage velocity and water head in saturated soil [5]. The equation is defined as follows:

(1) $$\begin{array}{c} v_{w}=-k_{w}\frac{\partial h_{w}}{\partial y}\text{ ,} \end{array}$$

where *v*_*w*_ is the unit volume flow, *k*_*w*_ is the permeability coefficient, and *h*_*w*_ is the total head gradient.

In 1931, Richards obtained through experimental analysis that the permeability coefficient in unsaturated soil seepage is a function of soil volume moisture content. At this time, the permeability coefficient will no longer be a constant [6]. Lei [7] deduced the general governing equation of two-dimensional seepage based on Darcy's law, which is based on the conservation of fluid mass at a certain point in unit time in steady-state seepage, and obtained the following formula:

(2) $$\begin{array}{c} \frac{\partial }{\partial x}\left(k_{x}\frac{\partial h_{w}}{\partial_{x}}\right)+\frac{\partial }{\partial y}\left(k_{y}\frac{\partial h_{w}}{\partial_{y}}\right),\\ =m_{w}\rho_{w}g\frac{\partial h_{w}}{\partial t}, \end{array}$$

where *k*_*x*_, *k*_*y*_ are the permeability coefficients in *x* and *y* directions, *m*_*w*_ is the specific water bulk density, *h*_*w*_ is the total head gradient, *ρ*_*w*_ is the density of water, and *g* is the acceleration of gravity.

*m*_*w*_=−∂*θ*/∂(*u*_*a*_ − *u*_*w*_), where (*u*_*a*_ − *u*_*w*_) denotes the matrix suction and *θ* denotes the volumetric water content.

### 2.2. Unsaturated Soil Shear Strength Equation

Based on the shear strength theory [8] proposed by Coulomb in 1773, many scholars at home and abroad have put forward numerous theoretical formulas for the shear strength of unsaturated soils through experiments and experience over the years. The commonly used shear strength formula of unsaturated soil is proposed by Fredlund et al. [9]. On the basis of unsaturated soil mechanics, the shear strength formula is expressed as a function of net stress, and matrix suction is defined as follows:

(3) $$\begin{array}{c} \tau =c{}^{\prime }+\left(\sigma -u_{a}\right)\text{tan}\varphi {}^{\prime }+\left(u_{a}-u_{w}\right)\text{tan}\varphi^{b}, \end{array}$$

where *τ* represents the shear strength; *c*′ represents the cohesion and *φ*′ represents the internal friction angle, while both are strength parameters of soil;  (*σ* − *u*_*a*_) is the net normal stress,  (*u*_*a*_ − *u*_*w*_) is the matrix suction, and  *φ*^*b*^ is the internal friction angle of suction.

In the strength theory proposed by Fredlund, the value of *φ*^*b*^ has always been difficult and cannot be directly applied in specific cases. In order to solve this problem, scholars began to consider linking this parameter with the soil-water characteristic curve. Vanapalli et al. [10] proposed the Vanapalli strength theory formula through a large amount of experimental data as follows:

(4) $$\begin{array}{c} \tau =c{}^{\prime }+\left(\sigma -u_{a}\right)\text{tan}\varphi {}^{\prime }+\left(u_{a}-u_{w}\right)\;\frac{\theta_{w}-\theta_{r}}{\theta_{s}-\theta_{r}}\text{tan}\varphi {}^{\prime }\text{,} \end{array}$$

where *θ*_*w*_ is the soil-water content, *θ*_*r*_ is the residual moisture content, and *θ*_*s*_ is the saturated volumetric water content.

### 2.3. Expression of Permeability Coefficient of Unsaturated Soil

In unsaturated soil, the permeability coefficient is a function of the volumetric water content of soil, so there must be a relationship between them. Fredlund et al. [11] and Vanapalli et al. [12] explored the relationship between the two and found that there is a functional relationship called soil-water characteristic curve between the matrix suction and the volumetric water content, and this curve can be used to better evaluate of engineering mechanical properties of unsaturated soils. In the soil-water characteristic curve, the matrix suction decreases with the increase of volume moisture content. According to the soil-water characteristic curve, the relationship between the permeability coefficient of unsaturated soil and matrix suction can be determined. In the numerical simulation, the parameters can be set according to the relationship between them to simulate the rainfall infiltration of unsaturated soil. Studying the interaction relationship between moisture content and the mechanical properties of unsaturated soils can realize the analysis of the influence of the internal pore water pressure and saturation of the subgrade in Africa on the overall stability of the subgrade in seasonally flooded subgrades.

It can be seen from the above that, in unsaturated soil, soil moisture content and matrix suction, permeability coefficient and matrix suction, permeability coefficient, and moisture content are closely related. Generally speaking, it is assumed that the deformation will not occur in the skeleton of soil; that is, the permeability coefficient of soil will not be affected by the change of pore volume between soil particles. At this time, the saturation of the soil is the primary factor affecting the permeability coefficient. In ABAQUS, saturation (*S*_*r*_) is defined as an independent variable, permeability coefficient (*k*_*w*_) and matrix suction {(*u*_*a*_ − *u*_*w*_)} are dependent variables, and the permeability coefficient of unsaturated soil is derived from the relationship curve between saturation and matrix suction. The relationship in [13] is as follows:

(5) $$\begin{array}{c} k_{w}=\frac{a_{w}k_{ws}}{\left[a_{w}+{\left(b_{w}\times \left(u_{a}-u_{w}\right)\right)}^{c_{w}}\right]}, \end{array}$$

where *k*_*w*_ is the permeability coefficient of unsaturated soil, *k*_*ws*_ is the saturated permeability coefficient of fill under actual compaction, *u*_*a*_ is the unsaturated rustic pressure, *u*_*w*_ is the unsaturated soil-water pressure, and *a*_*w*_, *b*_*w*_, and *c*_*w*_ are material factors.

(6) $$\begin{array}{c} S_{r}=S_{i}+\left(S_{n}-S_{i}\right)\frac{a_{s}}{\left[a_{s}+{\left(b_{s}\times \left(u_{a}-u_{w}\right)\right)}^{c_{s}}\right]}, \end{array}$$

where *S*_*r*_is the saturation. *S*_*i*_ is the residual saturation, *S*_*n*_ is the maximum saturation, and *a*_*s*_, *b*_*s*_, and *c*_*s*_ are material factors.

### 2.4. ABAQUS Strength Reduction Method

This paper uses the strength reduction method to analyze the high liquid limit red clay roadbed in Africa and explores the changing law of the safety factor of the slope after rainfall. The strength reduction method was first proposed by Zienkiewicz et al. [14] in 1975, and the method was described as artificially assigning a series of reduction coefficients based on the actual material strength parameters of the slope and gradually weakening through the continuous increase of the strength parameters. Its shear strength makes the slope reach a critical failure state. The reduced cohesion (*c*_*m*_) is expressed as the ratio of the actual cohesion (*c*) to the reduction coefficient (*F*_*r*_). The reduced internal friction angle (*φ*_*m*_) is expressed as the inverse tangent of the ratio of the tangent value of the actual internal friction angle (tan*φ*) to the reduction coefficient (*F*_*r*_). Among them, the reduction factor when a critical failure occurs on the slope is the safety factor (*F*_*s*_) of the slope. The expression equation is shown in the following formulas:

(7) $$\begin{array}{c} c_{m}=\frac{c}{F_{r}}, \end{array}$$

(8) $$\begin{array}{c} \varphi_{m}=\text{arctan}\left(\frac{\text{tan}\varphi }{F_{r}}\right), \end{array}$$

where *c* and *φ* are the shear strength parameters that the soil can provide, *c*_*m*_ and *φ*_*m*_ are the shear strength parameters after reduction, and *F*_*r*_ is the strength reduction factor.

The realization of the strength reduction method in the software is to give the soil material the initial shear strength, then set the field variable as the reduction factor, and continuously increase the reduction factor of the slope to make the slope reach critical failure. The value of the field variable at this time is the value of the safety factor of the slope, and the safety factor in the numerical calculation can be used as a criterion for assessing whether the roadbed is stable.

## 3. Numerical Simulation of Steady-State Seepage Flow of Seasonally Flooded Subgrade in Africa

Combined with the above theoretical analysis, use the software provided coupled pore fluid flow and stress analysis to simulate saturated-unsaturated soil. Taking the cross section on the left side of PK5 + 500 in the project as an example, the width of the roadbed is 33.5 m, the height of the roadbed is 8 m, the slope ratio is 1 : 1.5, and the initial groundwater level is −2 m. According to Zhao [15], the annual average rainfall of the project location is more than 4650 mm, of which the rainy season lasts for 8 months and the dry season lasts for 4 months.

**Figure 1:**
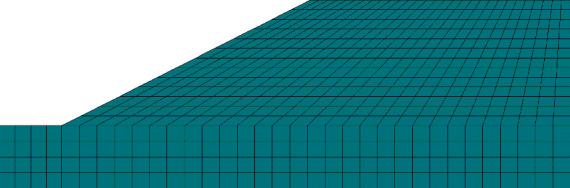
Two-dimensional model and grid division of the submerged roadbed.

The meteorological department is divided into six levels according to the total rainfall of 24 hours. In this simulation, the selected rainfall duration is set to 72 hours, the rainfall intensity is 50 mm/d (heavy rain) for rainfall infiltration simulation, and then we establish a two-dimensional finite element model of the red clay roadbed as shown in Figure 1.

### 3.1. Material and Parameter Setting

The basic physical properties of undisturbed red clay can be obtained through a test, as shown in Table 1. The density is measured by the ring shear testing, the cohesion and the angle of internal friction are measured by the direct shear test, and the permeability coefficient is measured by the variable water level method.

**Figure 2:**
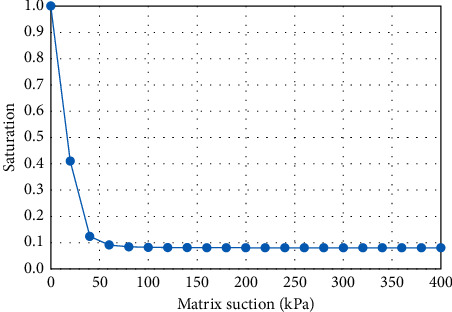
Soil-water characteristic curve.

**Figure 3:**
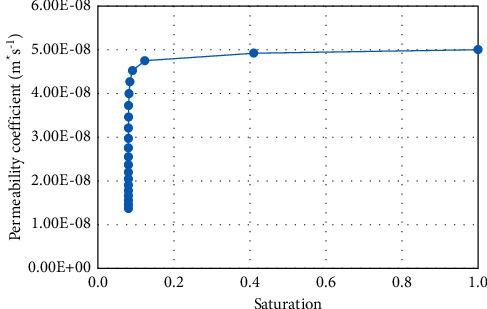
Moisture absorption curve of red clay.

Due to the lack of roadbed filling material at the project site, red clay is used for direct filling. The saturated permeability coefficient of red clay is 5 × 10^−8^ m/s. According to formulas (5) and (6), the change curve of matrix suction of red clay with saturation (Figure 2) and the change of permeability coefficient with saturation can be obtained curve (Figure 3). Inside of the formula, *a*_*w*_, *b*_*w*_, and *c*_*w*_ are 1000, 0.01, and 1.7; *S*_*i*_ denotes the residual saturation, and we take the number of 0.08;  *S*_*n*_ denotes the maximum saturation, and we take the number of 1;  *a*_*s*_, *b*_*s*_, and *c*_*s*_ are also used as material parameters with the numbers of 1, 5*e*-5, and 3.5.

### 3.2. Head Boundary Condition Setting

(i) Setting of pore water pressure boundary conditions: in this example, a boundary condition of pore water pressure of 0 is set at −2 m underground to establish the pore pressure space function along the linear section of the height as follows:
  (9) $$\begin{array}{c} u_{w}=\left(H_{1}-z\right)\gamma_{w}, \end{array}$$
where *u*_*w*_ is the pore water pressure (KPa); *H*_1_ is the total head (m); *z* is the immersion height (m); *γ*_*w*_ is the water severity (N/m^3^).
(ii) Impermeable boundary condition: the flow through the boundary condition is 0, ABAQUS defaults all boundary conditions at the boundary to 0, and no additional settings are required in this model.

### 3.3. Setting of Strength Reduction Factor

When intensity reduction is carried out in the model after rainfall, the internal friction angle and cohesion of red clay changing with field variables should be set in the property module of ABAQUS, where the field variables are mentioned in Section 2.4, as shown in Table 2. The relationship between the internal friction angle and the reduction coefficient in the table is expressed as *ϕ*_*m*_=arctan(tan*ϕ*/*F*_*r*_), where *ϕ*_*m*_ is the internal friction angle after reduction, *ϕ* is the actual internal friction angle, and *F*_*r*_ is the reduction coefficient. The relationship between cohesion and reduction coefficient is expressed as *c*_*m*_=*c*/*F*_*r*_, where *c*_*m*_ is the cohesion after reduction and *c* is the actual cohesion.

### 3.4. Numerical Simulation Calculation Process

**Figure 4:**
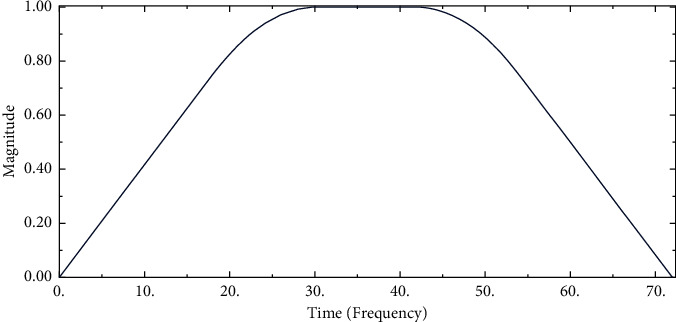
Rainfall amplitude curve.

Rainfall infiltration analysis: according to the information provided in Sections 2.1 and 2.2, first establish the red clay subgrade slope model, then give the above material properties to the model, then establish the soil analysis step called initial, divide the grid, and finally calculate the initial state of the subgrade. We save the calculated model and results of the initial state of the roadbed and then add a soil analysis step named infiltration on the basis of the saved model, which is obtained by defining the rainfall amplitude curve (shown in Table 3 and Figure 4) and setting the rainfall boundary conditions as the final rainfall infiltration model.

Strength reduction: save and extract the pore water pressure of each node in the results of the above rainfall infiltration model, convert it into the load imposed on each node, and finally select an appropriate incremental step for strength reduction analysis as needed.

## 4. Analysis of Rainfall Infiltration Results

This section is divided into two parts for analysis. First, the initial state of the roadbed is analyzed, and the pore water pressure and initial saturation distribution cloud diagram under the initial state are obtained. Then, the analysis of rainfall infiltration is performed by adding the infiltration analysis steps and rainfall, and finally, the stress field and displacement field of the red clay roadbed under rainfall conditions are obtained.

### 4.1. Initial Status Analysis

**Figure 5:**
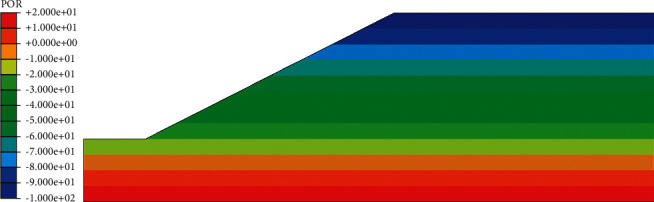
Cloud map of pore water pressure before rainfall.

**Figure 6:**
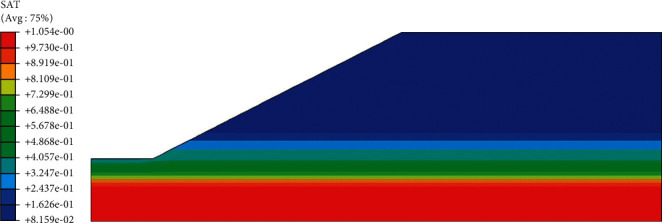
Saturation cloud map before rainfall.

**Figure 7:**
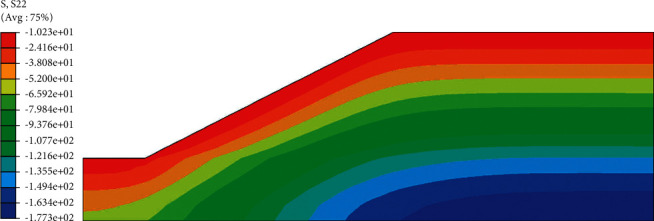
Vertical effective stress cloud map before rainfall.

According to the above boundary conditions, the initial distribution of pore water pressure and saturation of red clay subgrade before rainfall can be obtained, as shown in Figures 5 and 6. It can be seen from the distribution cloud diagram that the water level is the boundary line inside the roadbed, the upper part is the unsaturated zone, and the pore water pressure is negative; the lower part is the saturated zone, and the pore water pressure is positive. In the vertical effective stress distribution diagram (Figure 7), the effective stress value of the slope top is 10.23 kPa, not 0, and from the distribution diagram, the vertical effective stress gradually increases from the outside to the inside. These phenomena show that there is negative pore water pressure in the subgrade, that is, matrix suction.

### 4.2. Seepage Field Analysis

**Figure 8:**
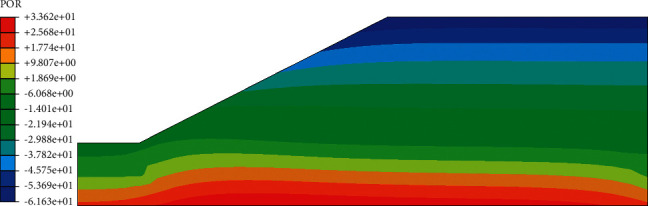
Cloud map of pore water pressure after 72 hours of rainfall.

**Figure 9:**
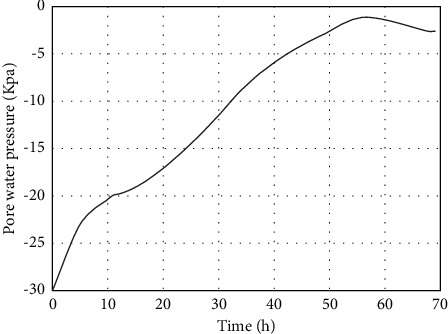
Pore pressure versus time curve.

**Figure 10:**
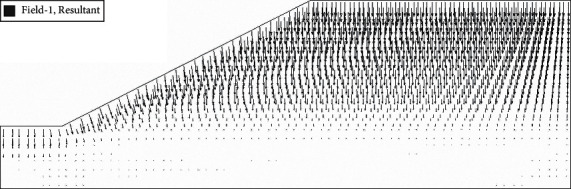
Flow rate incremental graph.

In the analysis of this chapter, the junction point of the slope foot (Figures 5 and 8) is selected as the comparison position before and after. It can be seen from the curve of pore pressure changing with time (Figure 9) that the pore pressure at the foot of slope was −30 kPa (0 h) before the rainfall, and the pore water pressure increased first, then decreased, and gradually tended to be stable. The pore pressure increases sharply from 0 h to 10 h. The reason for this phenomenon is that the matrix suction (negative pore water pressure) at the foot of the slope at the beginning of the rainfall is large, the rainwater quickly penetrates into the slope, the slope runoff is also converging towards the foot of the slope, causing a large amount of rainwater to infiltrate, and the pore pressure increases rapidly. At this time, the slope is 0.965; from 10 h to 56 h, the growth rate of pore pressure slows down, and the infiltration of rainwater causes the pore water pressure at the slope to gradually increase. The suction of the matrix gradually decreases, thereby slowing down the infiltration rate of rainwater into the roadbed. At this time, the slope is 0.418, the rainfall gradually stops at 56 h–72 h, the water inside the roadbed gradually seeps into the foundation, and the soil is deformed. The water is squeezed out, and the pore pressure is slightly reduced until it is basically unchanged. According to the rainfall amplitude curve in Figure 4, it can be seen that, at = 10.12 h, the rainwater is increasing. With the continuous infiltration of rainwater, the rainwater will gradually infiltrate into the subgrade. This phenomenon can be clearly seen in the velocity increment diagram (Figure 10).

### 4.3. Stress Field Analysis

**Figure 11:**
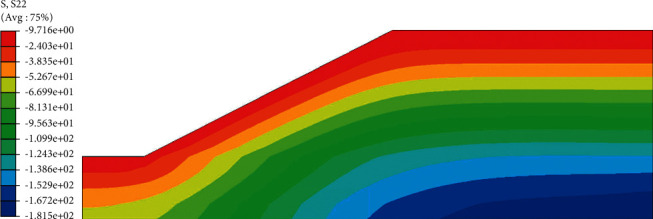
Effective stress cloud diagram after 72 hours of rainfall.

The effective stress distribution after rainfall (Figure 11) also showed a gradual increase from the outside to the inside. In the analysis of the initial state of the roadbed, the effective stress value at the top of the slope was 10.23 kPa, and the effective stress value at the top of the slope after 72 h rainfall was 9.7 kPa, a decrease of 5.2%. The main reason for the attenuation of the strength of the soil material is that the rainfall increases the saturation of the slope top soil, the suction of the matrix decreases, and the effective stress also decreases. The reduction of effective stress will lead to the decrease of roadbed stability and further affect the safety of the use of roadbed. Therefore, special attention should be paid to the change of effective stress when setting protection measures so as to prevent it from happening before it happens.

## 5. Slope Stability Changes after Rainfall

### 5.1. Displacement Field Analysis

**Figure 12:**
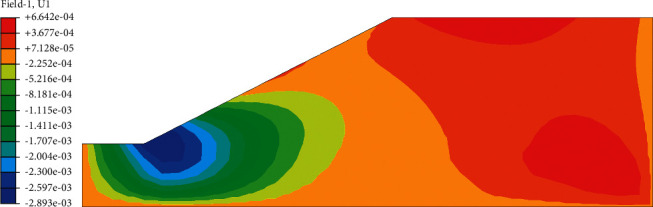
Incremental cloud map of horizontal displacement before and after rainfall.

**Figure 13:**
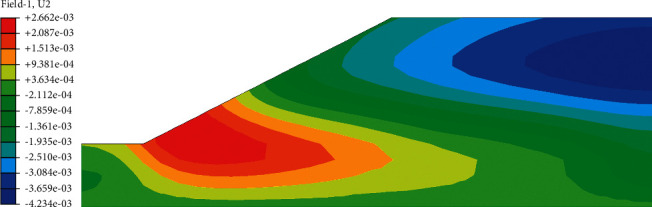
Incremental cloud map of vertical settlement before and after rainfall.

**Figure 14:**
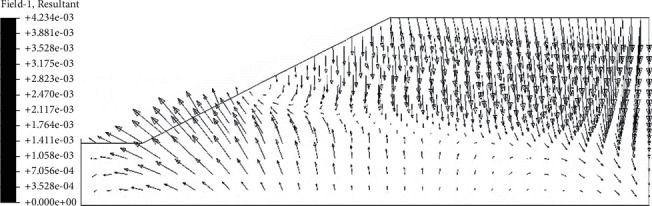
Displacement vector diagram after 72 h of rain.

After the analysis of slope seepage field and stress field in the above chapter, it can be concluded that the change of suction caused by rainwater infiltration is the key factor influencing the overall stability of roadbed by rainfall. Rainfall will cause displacement of roadbed slope, and displacement increment before and after rainfall can be obtained by field output module in ABAQUS. In the displacement increment cloud image, we can clearly see the location of the maximum horizontal displacement and maximum vertical settlement of the roadbed. In the nephogram of horizontal displacement increment (Figure 12), it can be seen intuitively that the maximum displacement occurs at the toe of the subgrade slope, and the increment is 2.89 mm. In the cloud diagram of vertical settlement increment (Figure 13), it can be seen that the maximum settlement occurs in the middle of the subgrade, and the increment is 4.23 mm. The above viewpoint can also be confirmed in the displacement vector diagram (Figure 14). It can be seen that the slope toe tends to slide outward, and there is the maximum settlement in the middle of the subgrade.

### 5.2. Safety Factor Analysis

**Figure 15:**
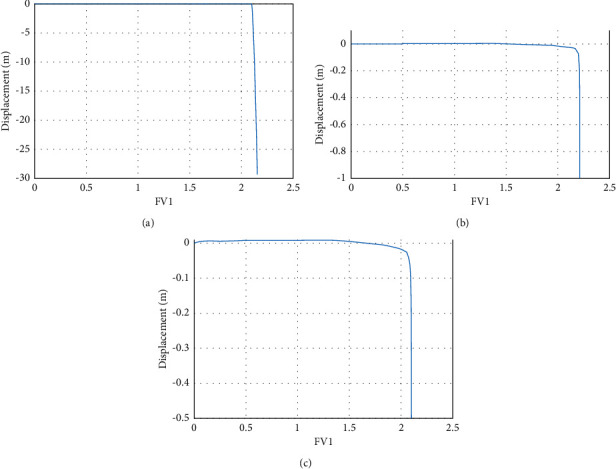
Curve diagram of safety factor under different conditions of roadbed. (a) Without groundwater safety factor curve diagram. (b) Curve of safety factor before rain. (c) Curve of safety factor after 72 hours of rain.

Continuous rainfall will not only affect the change of seepage field, displacement field, and stress field but also cause the change of slope safety factor. ABAQUS has two main principles for determining slope instability. The first method is to take the field variable corresponding to the turning point in the displacement curve (Figure 15), that is, the point of sudden displacement, as the safety factor. The second method is to take the field variable value corresponding to the step-by-step time as the safety factor of the subgrade when the plastic zone of the slope is connected in the cloud map. The values obtained by the two judgment methods are relatively similar. In this paper, the first method is selected as the evaluation standard for slope stability. The strength reduction method is used to analyze the safety factors of slopes in different states, as shown in Table 4. The safety factor after the rain is reduced by 11.6% compared to the safety factor of the roadbed without groundwater, and it is reduced by 4.9% compared to before the rain. It can be seen that rainfall has a significant impact on the overall stability of the roadbed.

**Figure 16:**
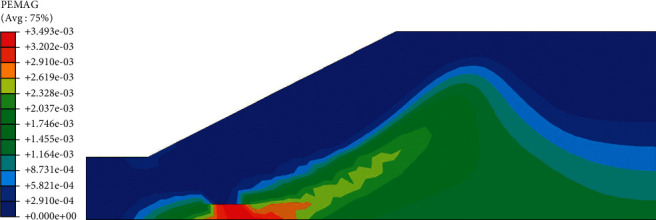
Cloud map of slope plastic zone before rainfall.

**Figure 17:**
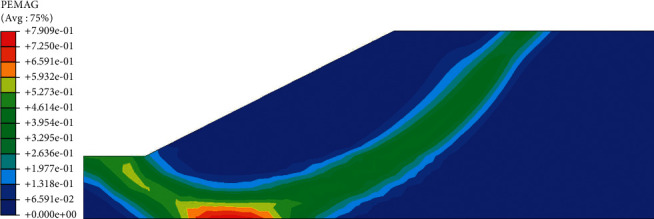
Cloud map of slope plastic zone after 72 hours of rainfall.

In the plastic zone cloud maps of the slope (Figures 16 and 17), the potential sliding surface of the slope can be clearly judged, showing a rough circular arc shape and indicating that the plastic zone will be transfixed when the slope is unstable. Combined with the curve of safety factor, it can be known that the slope displacement is very small when the slope begins to reduce. When the soil reaches the yield limit at the turning point, that is, when the plastic zone penetrates, the displacement will suddenly increase. Before the rainfall (Figure 16), the slope appears plastic zone at the bottom of the roadbed. The main reason is that the groundwater affects the strength parameters of the soil, which leads to the appearance of the plastic zone. As the rainfall continues, the plastic strain will extend from the foot of the slope to the top of the slope. After the rainfall ended, a penetrating plastic zone appeared inside the roadbed, and at this time, the slope was damaged and unstable.

## 6. Conclusions

1. When the high liquid limit red clay roadbed in Africa continues to rain in the rainy season, the rainwater will collect to the slope toe and continue to infiltrate into the roadbed. In this case, the groundwater level of the slope will continue to rise, and the internal saturation of the roadbed will gradually increase until it is saturated; the matrix suction will decrease with the increase of the saturation, and the strength parameters of the soil will also be degraded.
2. The rise of groundwater level caused by rainfall will reduce the effective stress of the subgrade as a whole, and a large horizontal displacement will occur at the foot of the slope. The plastic zone in the subgrade extends from the foot of the slope to the top of the slope and finally breaks through, forming a potential circular sliding surface inside the subgrade. The overall stability of the slope will decrease with the continuous rainfall, so special attention should be paid to the waterproof and drainage measures of the roadbed when the roadbed is designed.

## Figures and Tables

**Table 1:** Basic physical properties of red clay.

Material type	Density (g/cm^3^)	Cohesion (kPa)	Internal friction angle (°)	Permeability coefficient (m/s)	Poisson's ratio
Red clay	1.673	27.8	10	5 × 10^−8^	0.3

**Table 2:** Reduction factor settings.

Reduction factor	Cohesion (KPa)	Internal friction angle (°)
0.5	55.6	19.42540014
0.75	37.06666667	13.23012418
1	27.8	10
1.25	22.24	8.029256754
1.5	18.53333333	6.70442623
1.75	15.88571429	5.753605682
2	13.9	5.038368773
2.25	12.35555556	4.480971414
2.5	11.12	4.034435684
3	9.266666667	3.363727412

**Table 3:** Amplitude changes with time.

Time	Amplitude
0	0
24	1
36	1
72	0

**Table 4:** Safety factor of slope under different conditions.

Roadbed in different states	Without groundwater	Before the rain	After 72 h of rain
Safety factor (*F*_*r*_)	2.087	1.938	1.844

## Data Availability

The experimental data used to support the findings of this study are available from the corresponding author upon request.
